# Supplementary material for: A Norbornadiene-Based Molecular System for the Storage of Solar–Thermal Energy in an Aqueous Solution: Study of the Heat-Release Process Triggered by a Co(II)-Complex
Source: Molecules. 2023 Oct 25;28(21):7270. doi: 10.3390/molecules28217270 (PMC10650538; doi:10.3390/molecules28217270)
Supplement: Supplementary file 1 [file molecules-28-07270-s001.zip › molecules-2671516-supplementary.pdf]

SUPPLEMENTARY MATERIALS

A norbornadiene-based molecular system for the storage of solar-thermal energy in aqueous solution: study of the heat-release process triggered by a Co(II)-complex

**Figure S1**. Distribution diagrams of the species formed by HNBD1 (a) and HQC1 (b) as a function of pH in water calculated by using the determined p*K*a values.	S2
**Figure S2**. a) Evolution with time of the ^{1}H NMR spectrum (D_2O, pH 11, 298 K, 400 MHz) of $QC1^-$ at 370 K. b) Logarithmic decay of the integral of the ^{1}H NMR signal of $QC1^-$ at 2.5 ppm and corresponding fitting. Inset: Fitting details and parameters.	S3
**Figure S3:** UV-Vis spectra measured after each irradiation time of a 1.524 mM D_2O solution of $NBD1^-$ at pH = 10.96.	S4
**Figure S4**. ^{1}H NMR spectra measured after each irradiation period of a D_2O solution of $NBD1^-$ at pH = 10.67.	S5
**Figure S5.** a) Photon flux determination of the 275 nm LED used in this work. b) UV-Vis absorption spectra recorded during the photoisomerization experiments performed for the determination of quantum yield. c) Linear fit of the concentration of $NBD1^-$ calculated using absorbance values at 300 nm. Initial concentration of $NBD1^-$ 1.524 mM, pH of 10.96.	S6
**Figure S6** DSC thermograms for HNBD1. Scan rate 2 °C·min^{-1}.	S7
**Figure S7**. Example of ITC profile of the $QC1^-$ to $NBD1^-$ interconversion obtained upon addition of Co-TPPC (5% molar percentage relative to $QC1^-$) to an aqueous solution of $QC1^-$ (1.2 cm^3, 3.32 mM).	S8
**Figure S8**. ^{1}H NMR spectrum (400 MHz, $CDCl_3$) of (**3**).	S9
**Figure S9**. ^{13}C NMR spectrum (100 MHz, $CDCl_3$) of (**3**).	S10
**Figure S10**. ^{1}H NMR spectrum (400 MHz, $CDCl_3$) of HNBD1.	S11
**Figure S11**. ^{13}C NMR spectrum (100 MHz, $CDCl_3$) of HNBD1.	S12
**Figure S12**. ^{1}H NMR spectrum (400 MHz, $CDCl_3$) of HQC1.	S13
**Figure S13**. ^{13}C NMR spectrum (100 MHz, $CDCl_3$) of HQC1.	S14

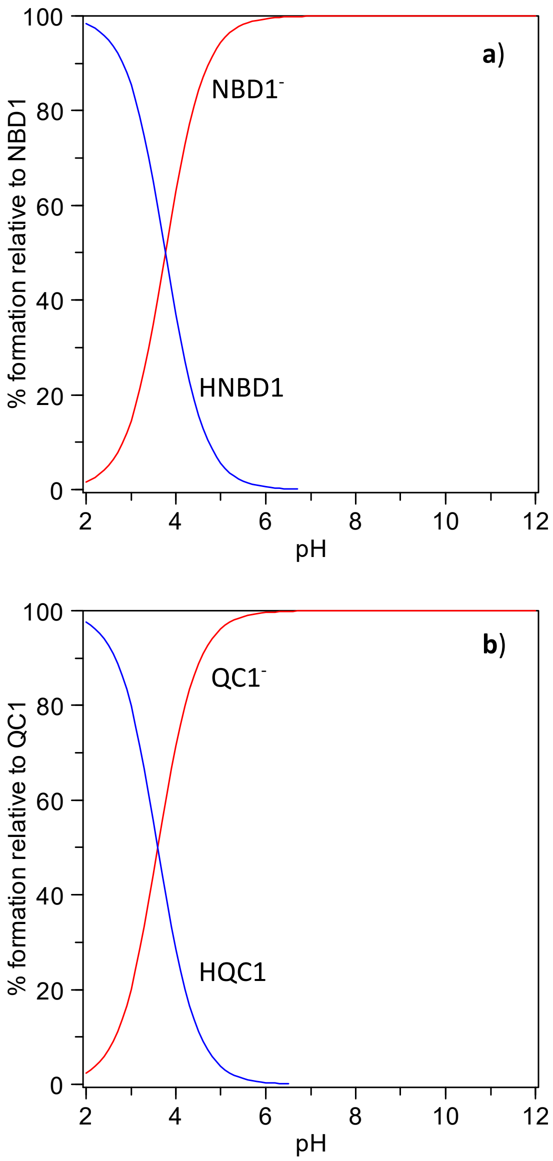

Figure S1. Distribution diagrams of the species formed by NBD1 (a) and QC1 (b) as a function of pH calculated by using the determined pK_a values.

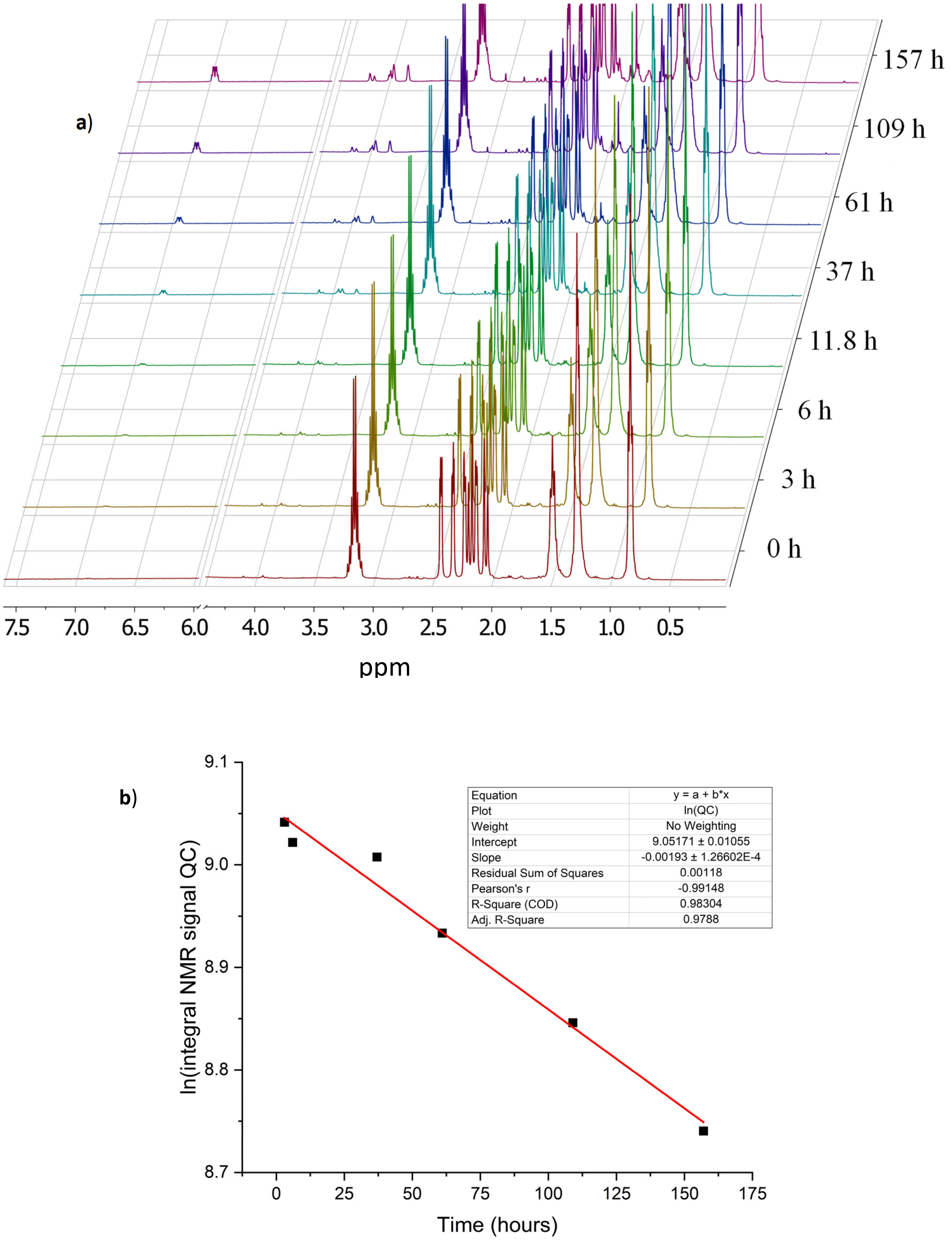

Figure S2. a) Evolution with time of the ^{1}H-NMR spectrum (D_2O, pH 11, 298 K, 400 MHz) of $QC1^{-}$ at 370 K. b) Logarithmic decay of the integral of the ^{1}H-NMR signal of $QD1^{-}$ at 2.5 ppm and corresponding fitting. Inset: Fitting details and parameters.

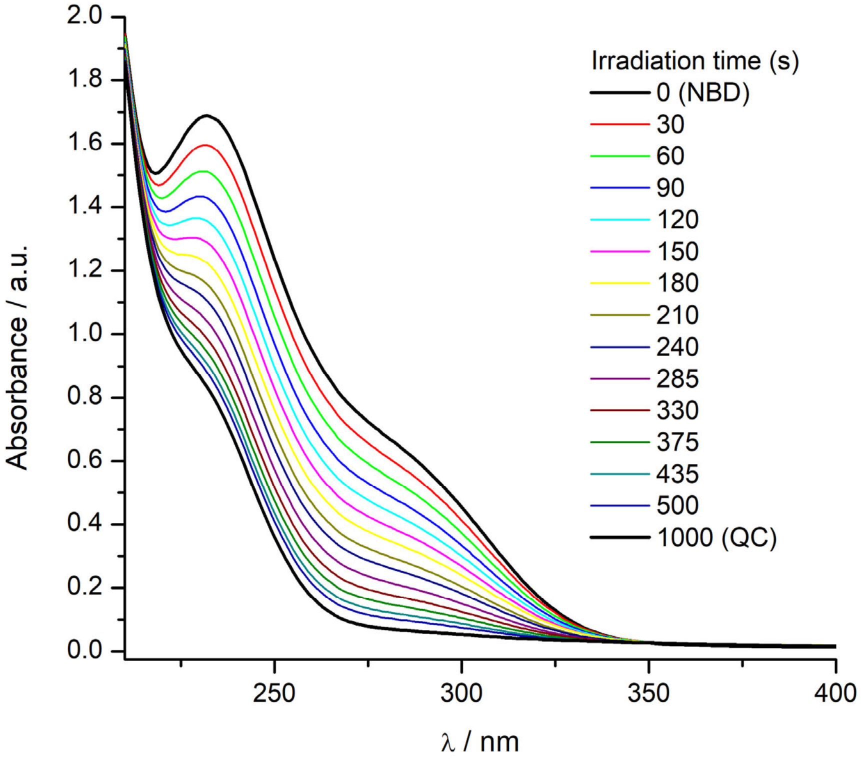

Figure S3: UV-Vis spectra measured after each irradiation time of a 1.524 mM D_2O solution of $NBD1^-$ at pH = 10.96.

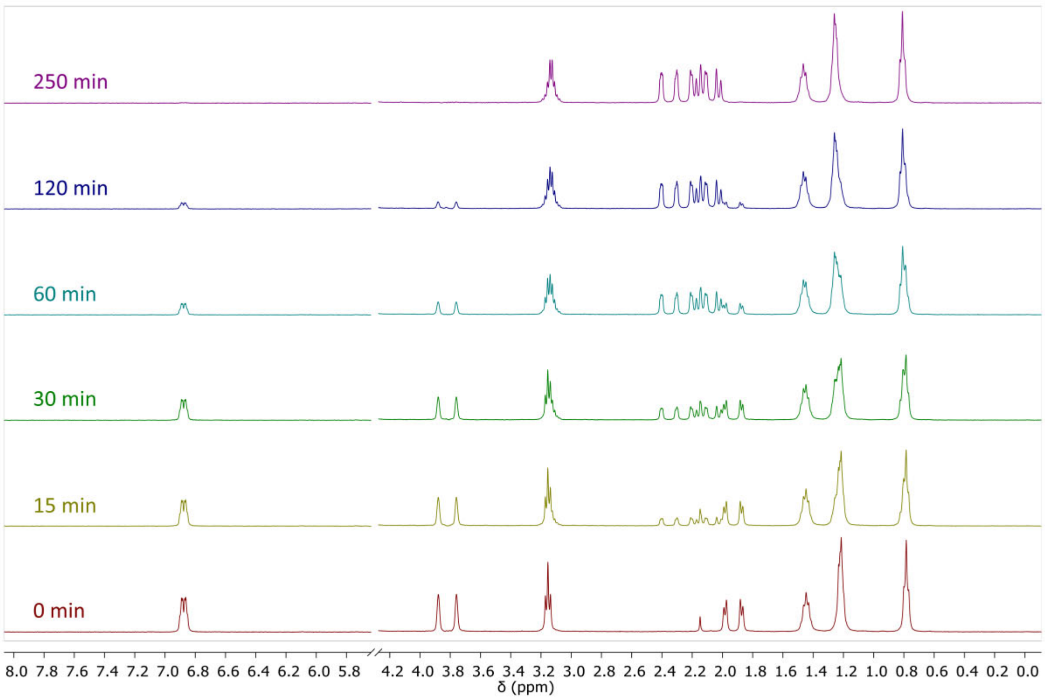

Figure S4. ^{1}H-NMR spectra measured after each irradiation period of a D_2O solution of $NBD1^-$ at pH = 10.67.

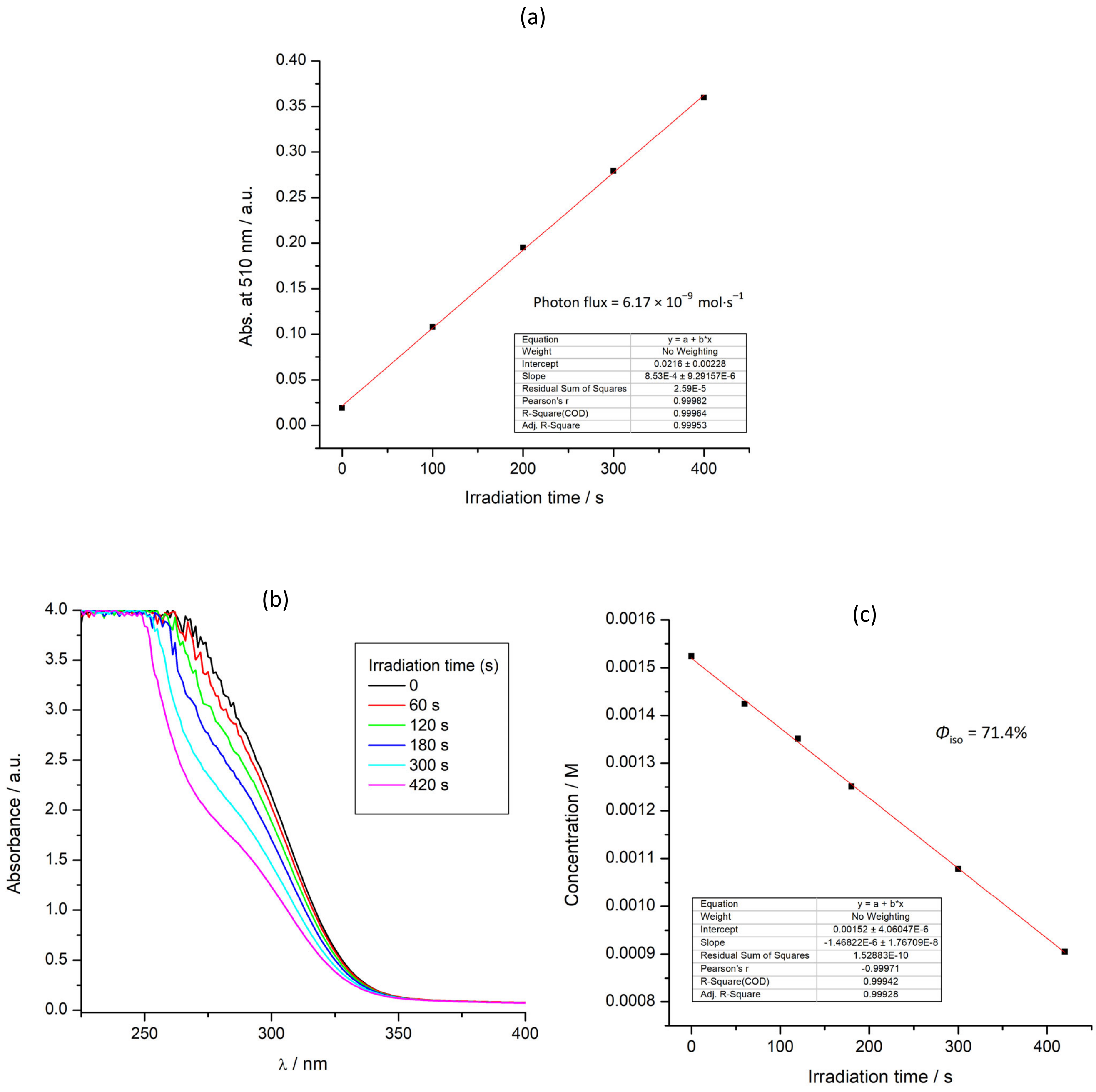

Figure S5. a) Photon flux determination of the 275 nm LED used in this work. b) UV-Vis absorption spectra recorded during the photoisomerization experiments performed for the determination of quantum yield. c) Linear fit of the concentration of $NBD1^-$ calculated using absorbance values at 300 nm. Initial concentration of $NBD1^-$ 1.524 mM, pH of 10.96.

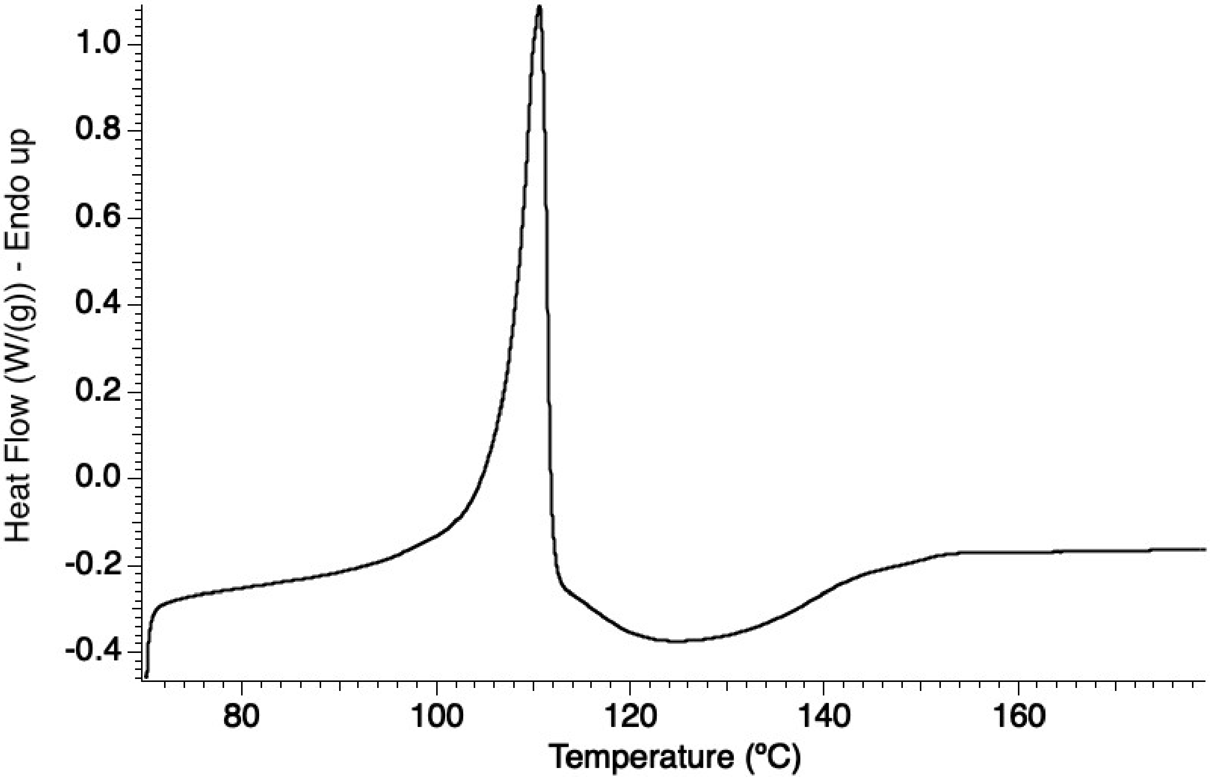

Figure S6. DSC thermograms for HNBD1. Scan rate 2 °C·min^{-1}.

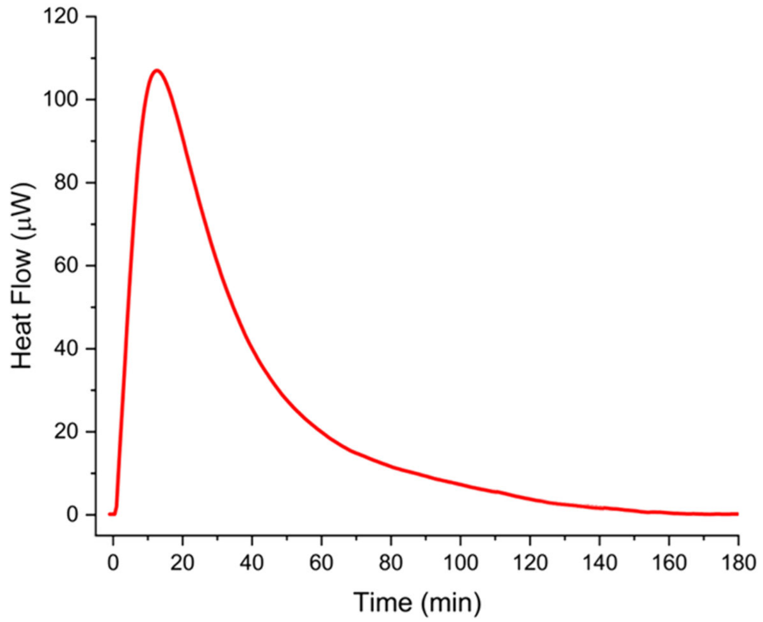

Figure S7. Example of ITC profile of the QC1$^-$ to NBD1$^-$ interconversion obtained upon addition of Co-TPPC (5% molar percentage relative to QC1$^-$) to an aqueous solution of QC1$^-$ (1.2 cm^3, 3.32 mM).

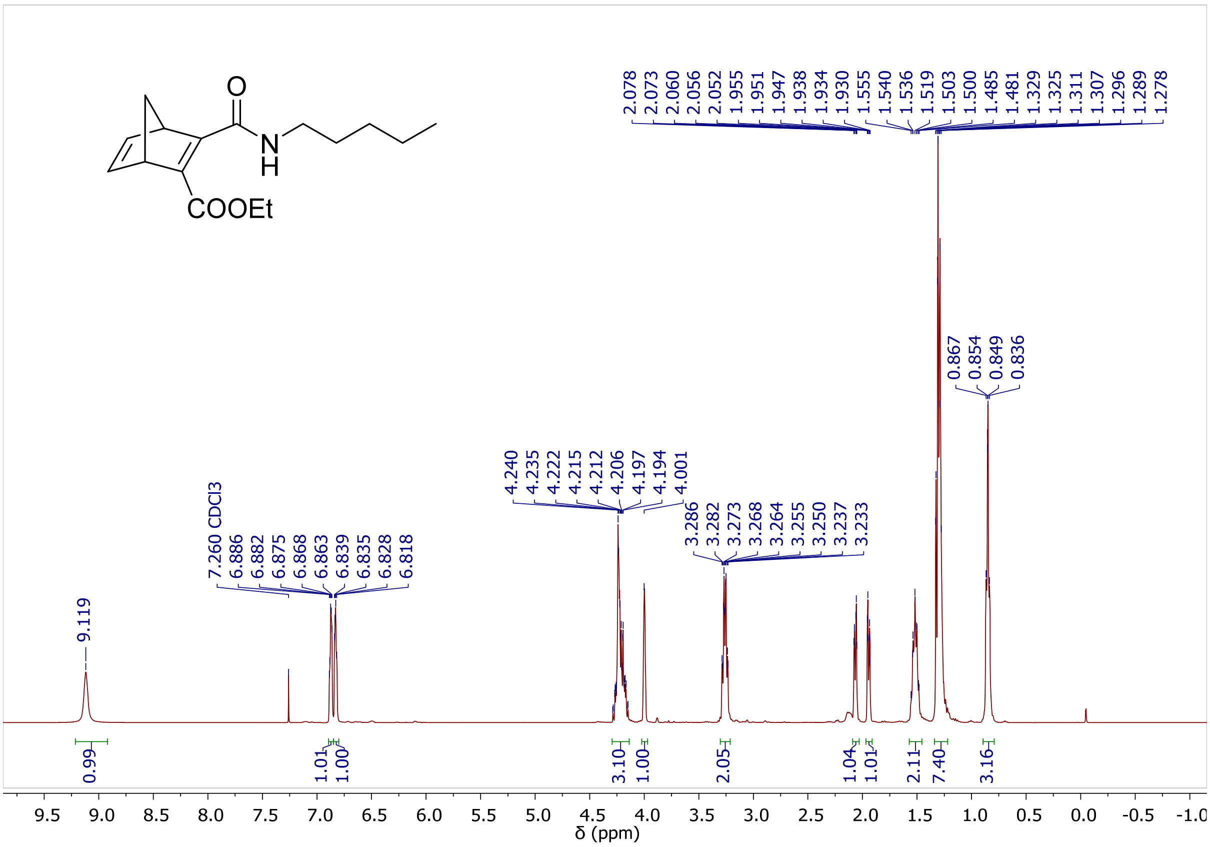

Figure S8. ^{1}H-NMR spectrum (400 MHz, $CDCl_3$) of (**3**).

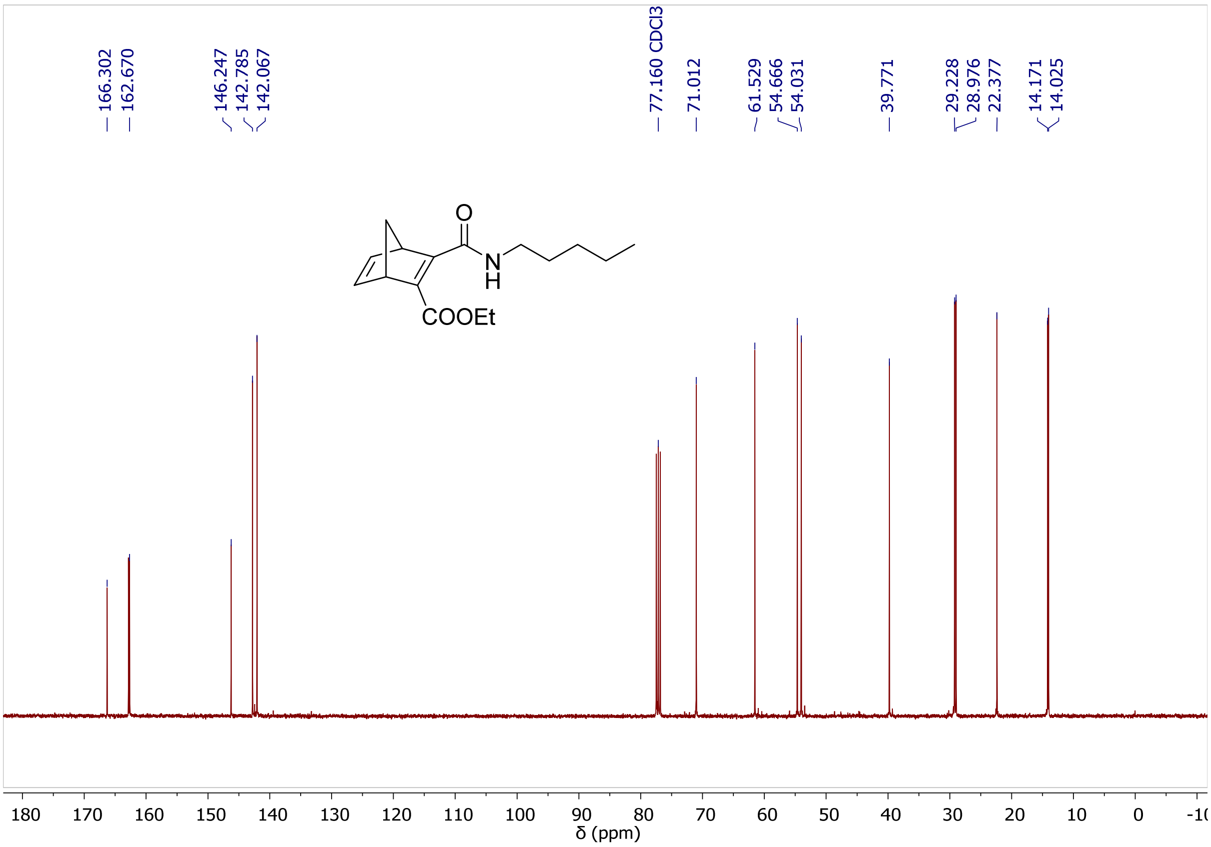

Figure S9. ^{13}C-NMR spectrum (100 MHz, $CDCl_3$) of (**3**).

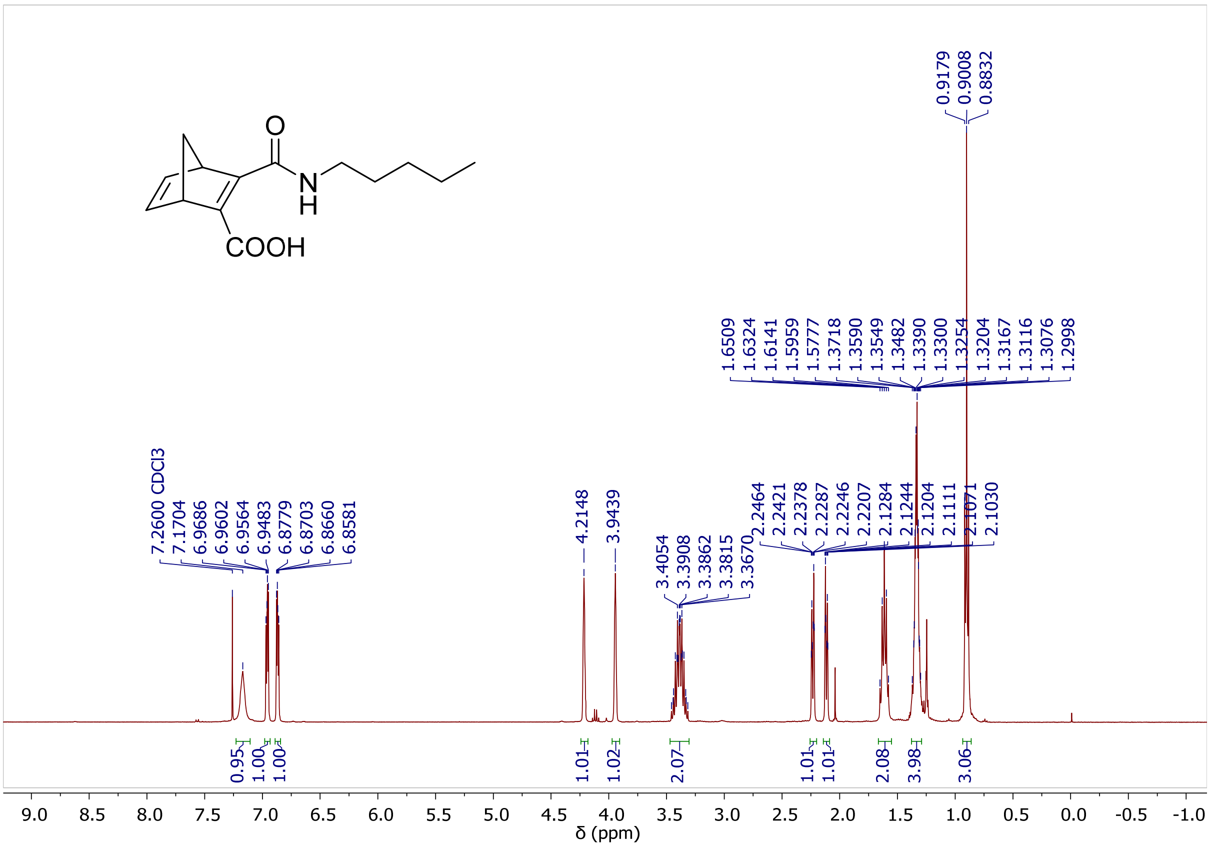

Figure S10. ^{1}H-NMR spectrum (400 MHz, $CDCl_3$) of HNBD1.

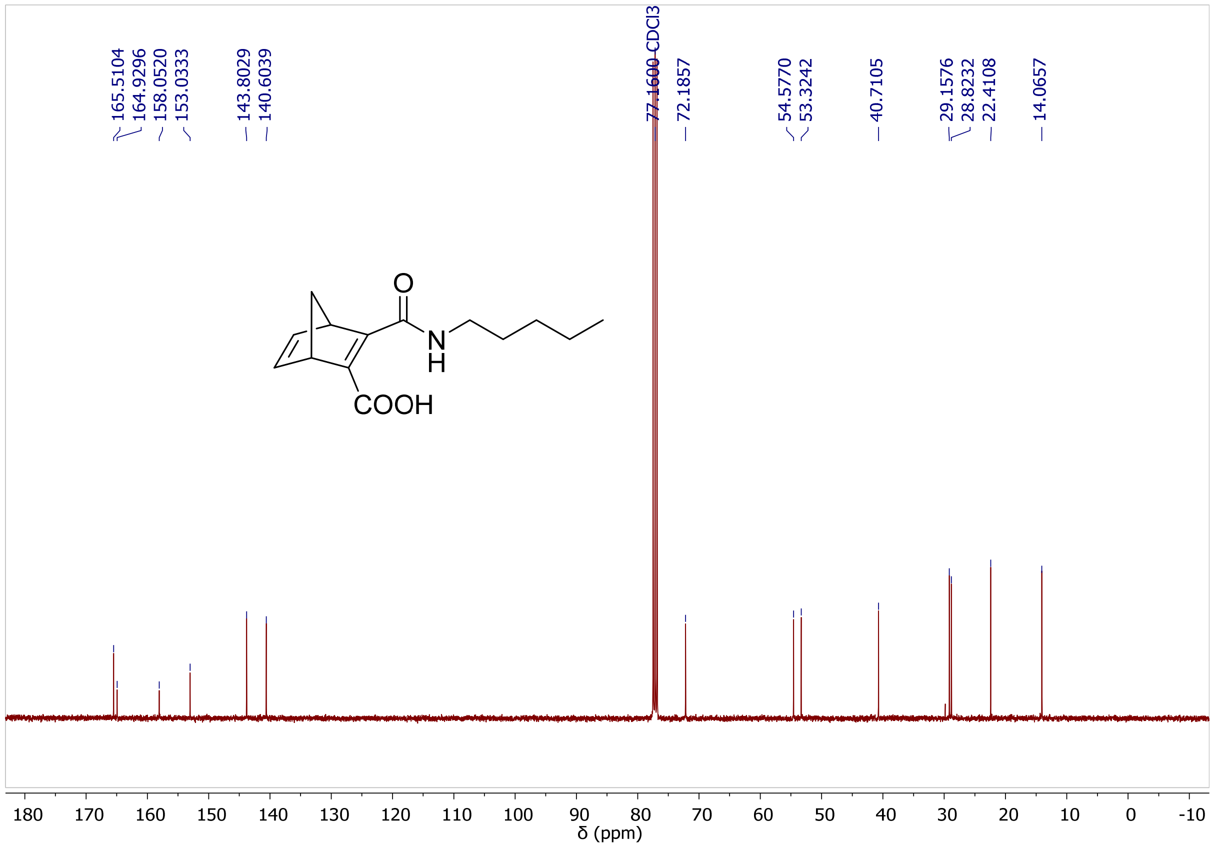

Figure S11. ^{13}C-NMR spectrum (100 MHz, $CDCl_3$) of HNBD1.

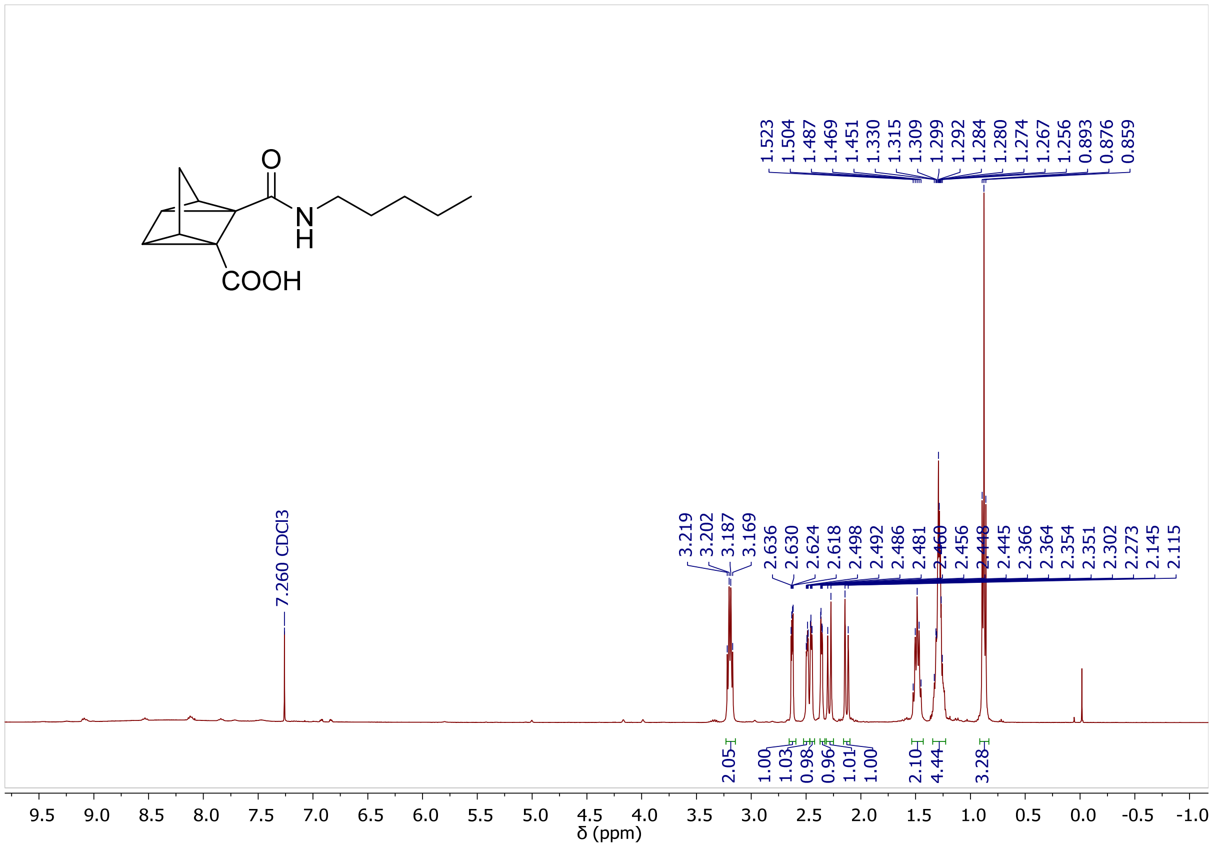

Figure S12. ^{1}H-NMR spectrum (400 MHz, $CDCl_3$) of HQC1.

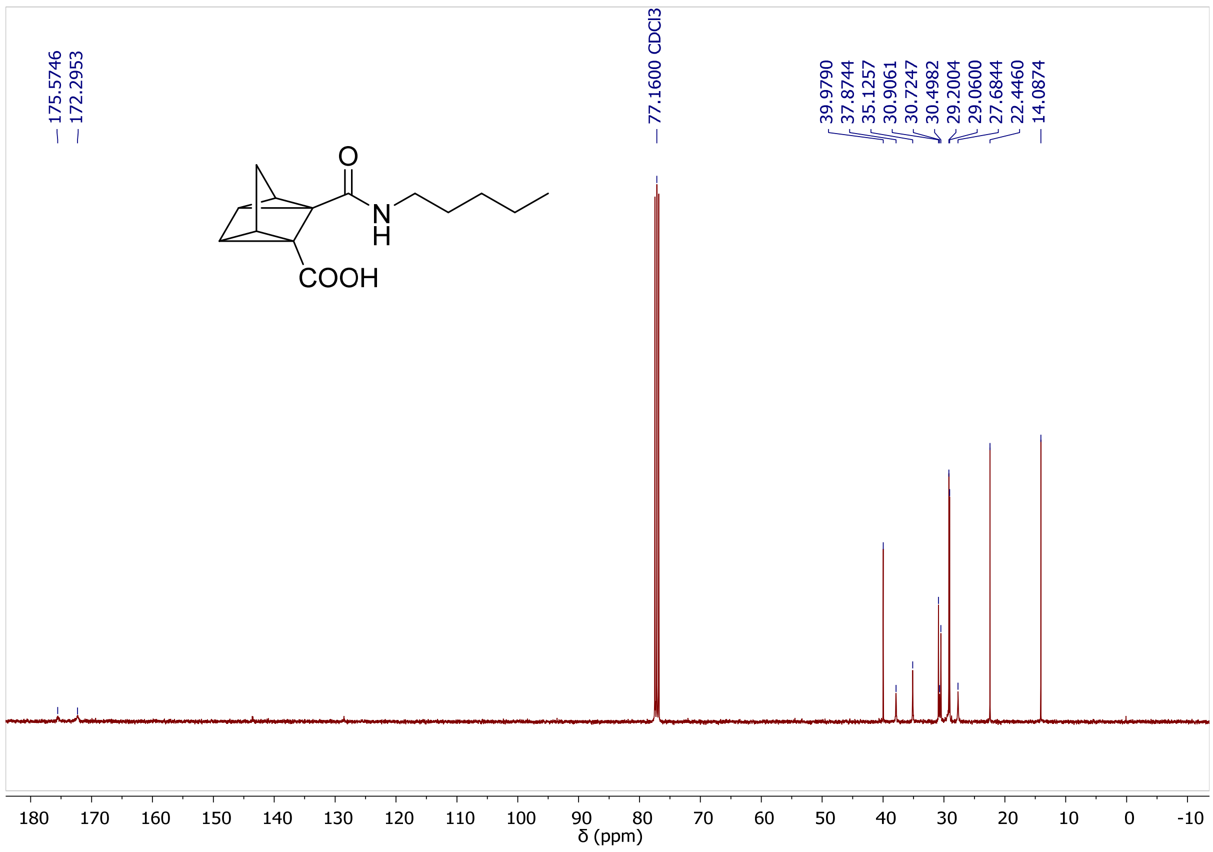

Figure S13. ^{13}C-NMR spectrum (100 MHz, $CDCl_3$) of HQC1.
